# Unraveling the impact of miRNAs on gouty arthritis: diagnostic significance and therapeutic opportunities

**DOI:** 10.1007/s00210-024-03603-9

**Published:** 2024-11-19

**Authors:** Sherif S. Abdel Mageed, Hanan Elimam, Ahmed E. Elesawy, Ahmed I. Abulsoud, Ahmed Amr Raouf, Manar Mohammed El Tabaa, Osama A. Mohammed, Mohamed Bakr Zaki, Mai A. Abd-Elmawla, Walaa A. El-Dakroury, Safwat Abdelhady Mangoura, Mahmoud A. Elrebehy, Mohammed S. Elballal, Aya A. Mohamed, Alaa Ashraf, Mustafa Ahmed Abdel-Reheim, Ali M. S. Eleragi, Hussein Abdellatif, Ahmed S. Doghish

**Affiliations:** 1 Pharmacology and Toxicology Department, Faculty of Pharmacy, Badr University in Cairo (BUC), Badr City, 11829 Cairo Egypt; 2 Biochemistry, Department of Biochemistry, Faculty of Pharmacy, University of Sadat City, Sadat City, 32897 Menoufia Egypt; 3 Department of Biochemistry, Faculty of Pharmacy, Badr University in Cairo (BUC), Badr City, 11829 Cairo Egypt; 4 Biochemistry Department, Faculty of Pharmacy, Heliopolis University, Cairo, 11785 Egypt; 5 Biochemistry and Molecular Biology Department, Faculty of Pharmacy (Boys), Al-Azhar University, Nasr City, 11231 Cairo Egypt; 6 Pharmacology & Environmental Toxicology, Environmental Studies & Research Institute (ESRI), University of Sadat City, Sadat City, 32897 Menoufia Egypt; 7 Department of Pharmacology, College of Medicine, University of Bisha, 61922 Bisha, Saudi Arabia; 8 Department of Biochemistry, Faculty of Pharmacy, Cairo University, Cairo, Egypt; 9 Department of Pharmaceutics and Industrial Pharmacy, Faculty of Pharmacy, Badr University in Cairo (BUC), Badr City, 11829 Cairo Egypt; 10 BK21 FOUR Team and Integrated Research Institute for Drug Development, College of Pharmacy, Dongguk University, Goyang, Republic of Korea; 11 Department of Pharmacognosy, Faculty of Pharmacy, Badr University in Cairo (BUC), Badr City, 11829 Cairo Egypt; 12 Department of Clinical Pharmacy and Pharmacy Practice, Faculty of Pharmacy, Badr University in Cairo (BUC), Badr City, 11829 Cairo Egypt; 13 Department of Pharmacology, College of Pharmacy, Shaqra University, 11961 Shaqra, Saudi Arabia; 14 Department of Microorganisms and Clinical Parasitology, College of Medicine, University of Bisha, 61922 Bisha, Saudi Arabia; 15 Department of Human and Clinical Anatomy, College of Medicine and Health Sciences, Sultan Qaboos University, Muscat, Oman; 16 Department of Anatomy and Embryology, Faculty of Medicine, University of Mansoura, Mansoura, 35516 Egypt; 17 Department of Biochemistry, Faculty of Pharmacy, Galala University, New Galala City, 43713, Suez,, Egypt

**Keywords:** Gouty arthritis, miRNA, Biomarker, Diagnosis, Therapy

## Abstract

Gouty arthritis is a prevalent inflammatory illness. Gout attacks begin when there is an imbalance in the body’s uric acid metabolism, which leads to urate buildup and the development of the ailment. A family of conserved, short non-coding RNAs known as microRNAs (miRNAs) can regulate post-transcriptional protein synthesis by attaching to the 3′ untranslated region (UTR) of messenger RNA (mRNA). An increasing amount of research is pointing to miRNAs as potential players in several inflammatory diseases, including gouty arthritis. miRNAs may influence the progression of the disease by regulating immune function and inflammatory responses. This review mainly focused on miRNAs and how they contribute to gouty arthritis. It also looked at how miRNAs could be used as diagnostic, prognostic, and potential therapeutic targets.

## Introduction

Gouty arthritis (GA) affects 3.9% of Americans, with its prevalence rising to 5.2% among men and 2.7% among women (Haneklaus et al. 2013). Monosodium urate (MSU) crystal formation in the joint capsule, bone, bursa, and cartilage causes this uric acid metabolism disorder. This buildup damages joints and may affect other organs such as the liver and kidney (Perez-Ruiz et al. 2015; Elseweidy et al. 2022; Ramos and Goldfarb 2022). Gout is linked to metabolic comorbidities such as myocardial infarction, type 2 diabetes, chronic renal disease, and premature death (Roughley et al. 2015; Fisher et al. 2017). Managing gouty arthritis bouts involves reducing pain and joint inflammation. Glucocorticoids, non-steroidal anti-inflammatory medications, and others are used. Gout episodes can be prevented by treating hyperuricemia with uric acid-lowering medication (Richette et al. 2017; FitzGerald et al. 2020). Recurrent attacks of gouty arthritis occur despite improved treatments and a greater understanding of its pathophysiology (Tin et al. 2018).

MicroRNA (miRNA) is a tiny, non-coding RNA molecule that has a length of approximately 20 to 25 nucleotides. While miRNA sequences are often conserved, variations in mature miRNAs can arise from mutations in the miRNA genes or post-transcriptional changes, both within and between species (Salman et al. 2023). The first time it was found was in 1993 when it was found in Caenorhabditis worms (Cai et al. 2009). Pri-miRNAs are extended primary miRNAs that are initially transcribed in the nucleus. DiGeorge syndrome critical region 8 (DGCR8) has been recognized as a protein that acts as a key component of the microprocessor complex, which also includes the RNase III enzyme Drosha. Upon binding to pri-miRNAs, DGCR8 recruits Drosha, which cleaves the pri-miRNA hairpin structure to release the pre-miRNA. Following this, the pre-miRNAs are transported out of the nucleus and exported to the cytoplasm by forming a complex with Exportin 5 (Exp5). Additionally, an enzyme known as Dicer is responsible for further cleaving them in the cytoplasm, which ultimately results in the formation of mature miRNAs (Suzuki et al. 2015; Cheng et al. 2016). There is a significant degree of evolutionary conservation among microRNAs (Chen et al. 2017). The miRNAs can have a significant impact on gene expression and function through their interactions with the RNA-induced silencing complex (RISC). They typically bind to complementary sequences in the 3′ untranslated region (UTR) of target mRNA transcripts, known as miRNA response elements (MREs). This binding event leads to the recruitment of the RISC complex, which includes Argonaute (Ago) proteins and other cofactors. Once the miRNA-RISC complex is formed, it can exert its regulatory effects on the target gene through several mechanisms, like translational repression, mRNA destabilization, or transcript cleavage. Through these mechanisms, a single miRNA can potentially target and regulate the expression of multiple genes. Indeed, this allows miRNAs to fine-tune gene expression networks and play important roles in diverse biological processes, such as development, differentiation, proliferation, and apoptosis. Furthermore, numerous miRNAs can collaborate to regulate the same target gene in a manner that provides synergistic effects (Bronisz et al. 2020; Grasso et al. 2020). It is estimated that human cells contain more than 2500 known miRNAs. However, Londin et al. (2015) identified a total of 3707 novel miRNAs in the human genome (Londin et al. 2015). Hence, this number has increased over time as new miRNAs have been discovered through advancements in sequencing technologies, deep sequencing, and bioinformatics approaches (Ismail et al. 2024). There is still a lack of complete comprehension of the functions of miRNAs; nonetheless, relevant research has proven their involvement in several activities, including cell development, metabolism, and inflammation (Papanagnou et al. 2016).

Multiple prior studies showed that miRNAs are crucial to the etiology of inflammatory disorders, including gout (Abdelmonem et al. 2021; El-Boghdady et al. 2023; Kortam et al. 2023). Another study has shown that miRNAs play a major role in gouty arthritis onset (Luo et al. 2022). However, up until today, these assessments have been conducted using a single method. They have not added any further subjects. Thus, this paper examines how miRNAs govern gout progression through uric acid metabolism, conventional inflammatory signaling pathways, and bone degradation (Luo et al. 2022). Based on this, the current review studied miRNA as a therapeutic intervention target, gout diagnosis, and prognostic tool.

## miRNA biogenesis and functions

Non-coding RNAs (ncRNAs), like miRNAs, are essential for the control of gene expression in most eukaryotic species. Numerous studies have been conducted to explore the remarkable flexibility, complex regulatory networks, and significant influence of these regulating switches on various biological pathways (Cui et al. 2019; Li et al. 2022a). The miRNA genes, or gene clusters that are either exclusively or cooperatively assembled into miRNA molecules, are the DNA sequences that precede miRNAs. The location of miRNAs, whether they are in the introns or the intergenic regions, can help differentiate between the canonical and non-canonical miRNA biogenesis pathways. The miRNAs located within introns are more likely to be processed through the non-canonical mirtron pathway, while miRNAs located in intergenic regions are typically processed through the canonical pathway (Rodriguez et al. 2004; Olena and Patton 2010).

The first phase of the canonical process, primary miRNA (pri-miRNA), is the result of RNA polymerase II’s encoding of miRNA gene sequences in the canonical biogenesis pathway. The pri-miRNA is then converted into the precursor miRNA (pre-miRNA). The RNase III endonuclease Dicer must first digest the miRNA after it has entered the cytoplasm through Exportin 5. The functional effector complex is directed by matured miRNA in a single-stranded form to modify complementary RNA targets (Denli et al. 2004; Newman and Hammond 2010; Wei et al. 2021; Erturk et al. 2022; Shang et al. 2023; Elballal et al. 2024).

**Fig. 1:**
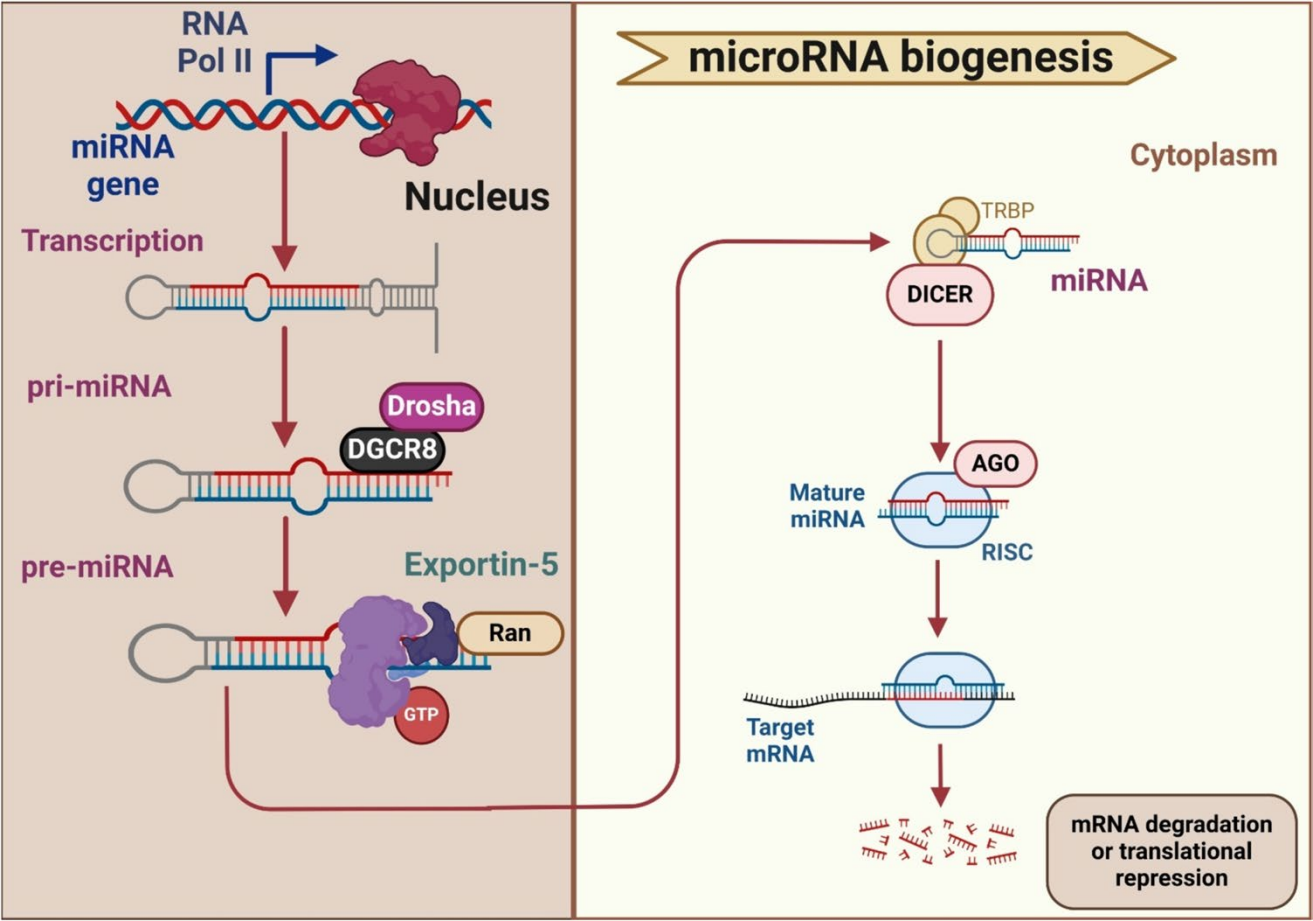
miRNA biogenesis. The pri-miRNA is converted into the precursor miRNA (pre-miRNA). The RNase III endonuclease Dicer must first digest the miRNA after it has entered the cytoplasm through Exportin 5. The functional effector complex is directed by matured miRNA in a single-stranded form to modify complementary RNA targets. Ago: argonaute1; DGCR8: DiGeorge Critical Region 8; Dicer: an endoribonuclease enzyme that in humans is encoded by the DICER1 gene; Drosha: double-stranded RNA-specific endoribonuclease; Ran: RAS-related nuclear protein; RISC: RNA-induced silencing complex; RNA Pol II: RNA polymerase II; TRBP: transactivation response element RNA-binding protein. This figure was created with BioRender (https://biorender.com/)

Only a handful of miRNAs undergo non-canonical processing. Researchers have made significant progress in understanding the intricate and varied processes of miRNA synthesis, processing, and maturation. They have achieved this through the utilization of essential procedures and approaches such as tRNase Z and Mirtrons, as well as dicer-independent approaches (Fig. 1) (Ruby et al. 2007; Babiarz et al. 2008; Ergin and Çetinkaya 2022).

Even though miRNAs have been there for a while, their crucial significance in an organism’s operation has just lately been recognized. There has been an overwhelming amount of research on miRNAs and their potential involvement in metabolic and inflammatory diseases, including gouty arthritis. Over one hundred distinct varieties of arthritis have been identified, and miRNAs have been found to have a role in some of these types (Papanagnou et al. 2016; Peng et al. 2023). miRNAs greatly aid in the control of an enormous variety of biological processes. These regulators may contribute to gout’s development and represent therapeutic targets. Because miRNAs regulate many mRNAs post-transcriptionally, their actions depend on their environment. These functions are affected by miRNA-gene communication, miRNA-target mRNA amount and affinities, cell type, expression of miRNA level, and subcellular miRNA expression (Luo et al. 2022).

Targeting the processing of miRNAs may provide new insights for the therapeutic strategies of gout in the future. miRNAs are implicated in the development of hyperuricemia and contribute significantly to the disease because of their involvement. In gouty arthritis, when macrophage inflammatory pathways are activated, the macrophages become differentiated for the M1 phenotype and produce significant quantities of pro-inflammatory molecules. miRNAs are also involved in this process and have a regulatory role. It has been demonstrated via a multitude of research that miRNAs have a role in the progression of bone degradation by inhibiting osteoclast proliferation and development (Luo et al. 2022).

To fully understand the complex biogenesis and wide range of functions of miRNAs, we need to know exactly what role they play in the pathophysiology of gouty arthritis. This is because these small RNA molecules are key to controlling the inflammatory processes and cell pathways that make up this condition.

## The pathophysiological mechanisms of gouty arthritis

Gout represents the most frequent cause of persistent inflammatory arthritis (Dalbeth et al. 2016; Vargas-Santos et al. 2016). Gout is a metabolic disorder that causes urate or uric acid to build up in tissues and blood. MSU crystals are created when the urate salts precipitate and tissues get supersaturated. Deposition of these crystals is most commonly reported in synovium, bone, skin, tendon, cartilage, ligament, and kidney. In addition, it involved a range of other musculoskeletal and non-musculoskeletal tissues (Towiwat et al. 2019).

The biochemical characteristic marker of gout is the urate saturation of extracellular fluid. The perception of urate will occur when its solubility limit in plasma or serum exceeds 6.8 mg/dL (about 400 micromole/L) (Loeb 1972). A higher risk of developing gout is correlated with the severity of hyperuricemia (Gustafsson and Unwin 2013). Urate is the uric acid salt. It is a waste product that is produced when RNA, DNA, and ATP are broken down as part of purine metabolism. Most uric acid circulates as the urate anion. The liver enzyme uricase, also known as urate oxidase, converts the slightly soluble uric acid into the highly excretable water-soluble allantoin. As a result, allantoin is excreted from the kidneys much more effectively than uric acid (Johnson et al. 2005). The final step in purine metabolism is the conversion of hypoxanthine to xanthine, which is followed by uric acid by xanthine oxidase and allantoin by uricase. The solubility of allantoin is far higher than that of uric acid. Due to genetic alterations, humans, other primates, giraffes, and dalmatians are unable to generate uricase (Choi et al. 2005; Tan et al. 2016). Genetic mutations may be responsible for the inactivation of the uricase gene (Tan et al. 2016). As a disease, gout has a complicated etiology that involves genetic risk factors, medical comorbidities, and dietary factors. It is believed to be an innate immune system-triggered systemic inflammatory illness (Ragab et al. 2017).

Inflammasomes and the release of pro-inflammatory cytokines are the primary sources of inflammation associated with gout. Tumor necrosis factor-α (TNF-α), certain interleukins, and the NOD-like receptor protein 3 (NLRP3) inflammasome are involved in the development of gout. Macrophages, neutrophils, and monocytes are involved in these inflammatory amplification cascades (Zeng et al. 2016; So and Martinon 2017).

Numerous factors contribute to human hyperuricemia, such as a genetic deficiency of uricase, the reabsorption of 90% of filtered uric acid, and the limited solubility of urate and MSU in body fluids (Abhishek et al. 2017). The kidney is a major target in the regulation of serum urate levels through urate release via urate transporters and the re-absorption of filtered uric acid. Humans are susceptible to hyperuricemia and gout due to anomalies in the urate transporter and a failure to convert urate to allantoin (Álvarez-Lario and Macarrón-Vicente 2010; Kratzer et al. 2014).

Urate excretion takes place via two pathways: the kidneys excrete two-thirds of its amount and the gastrointestinal tract (GIT) excretes the remaining urate. Reduced excretion of uric acid through the GIT due to reduced secretory function of the transporter ABCG2 causes a rise in blood uric acid levels and improved renal excretion (Ichida et al. 2012).

Because uric acid crystals are insoluble, certain membrane transporters are required for them to cross cell membranes. URAT1 and the organic anion transporters (OAT1 and OAT3) are the two transporters that are involved in the urate transporter/channel (URAT) (Enomoto and Endou 2005; Mandal and Mount 2015).

Renal excretion of uric acid occurs in four stages. Following the initial phase of uric acid passage by the Bowman’s capsule (glomerular filtration), almost all urates going through the proximal tubules are reabsorbed. Part of the reabsorbed uric acid is secreted during the third phase, which concludes with another reabsorption phase in the proximal tubules. Approximately 10% of urate that has been filtered via Bowman’s capsule is filtered as uric acid, with the remaining urate being reabsorbed by the body (Bobulescu and Moe 2012).

Moreover, consuming purine-rich foods—such as cooked or processed foods, particularly those derived from animals and seafood—is a major factor in raising the precursors of uric acid.

**Fig. 2:**
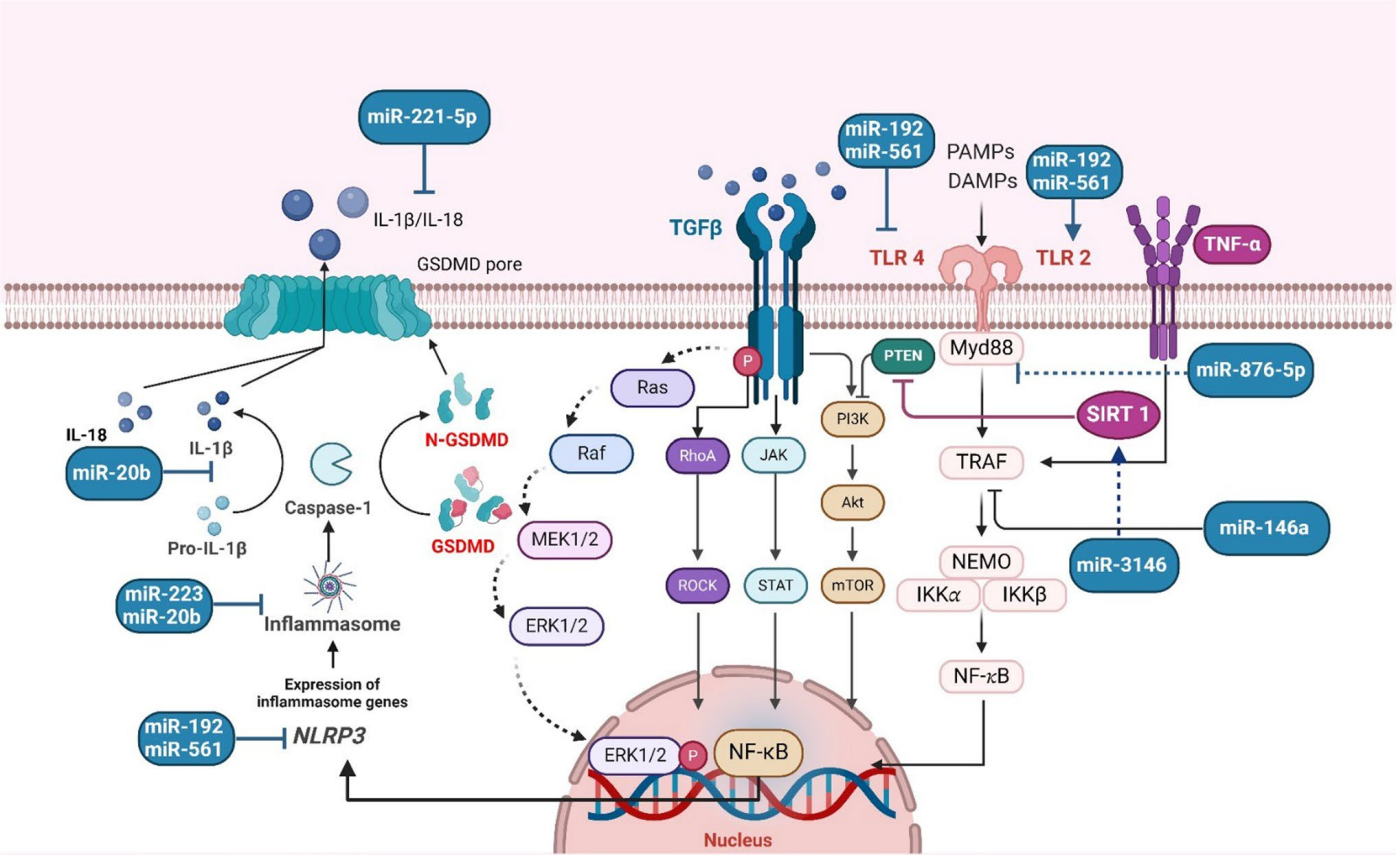
Molecular pathways involved in gouty arthritis and potential miRNA regulators. Inflammasomes and the release of pro-inflammatory cytokines are the primary sources of inflammation associated with gout. TNF-α, certain interleukins, and NLRP3 inflammasome are involved in the development of gout. Macrophages, neutrophils, and monocytes are involved in these inflammatory amplification cascades. AKT: protein kinase B; ERK: extracellular signal-regulated kinase; GSDMD: gasdermin D; IKK: IκB kinase; IL: interleukin; IRAK1: IL-1 receptor-associated kinase; JAK: Janus kinase; MEK: mitogen-activated extracellular signal-regulated kinase; mTOR: mammalian target of rapamycin; MyD88: myeloid differentiation primary-response protein 88; NEMO: nuclear factor-kappa B essential modulator; NLRP3: NOD-, LRR-, and pyrin domain-containing protein 3; NF-κB: nuclear factor-κB; PI3K: phosphatidyl inositol 3-kinase; PTEN: phosphatase and tensin homolog; Raf: rapidly accelerated fibrosarcoma; Ras: renin angiotensin system; RhoA: Ras homolog gene family member A; ROCK: rho kinase; SIRT1: silent information regulator sirtuin 1; STAT: signal transducer and activator of transcription; TLR: toll-like receptor; TNF-α: tumor necrosis factor-alpha; TRAF: TNF receptor-associated factor. This figure was created with BioRender (https://biorender.com/)

On the other hand, vegetable-based purine-rich foods like beans, lentils, mushrooms, peas, legumes, and dairy products do not increase your chance of developing gout or hyperuricemia (Kanbara and Seyama 2011; Towiwat and Li 2015) (Fig. 2).

After determining the primary pathophysiological mechanisms that are responsible for gouty arthritis, it is of the utmost importance to investigate how miRNAs influence the immunological and inflammatory signaling pathways that are involved. This is because these regulatory molecules play a crucial part in determining the inflammatory response that is associated with the disease.

## Role of miRNAs in the regulation of immunological and inflammatory signaling in gouty arthritis

There are four phases in the development of gout: asymptomatic hyperuricemia (HUA), acute gouty arthritis, intercritical gout, and chronic tophaceous gout (Pascart and Richette 2017). HUA is a prevalent clinical characteristic of gouty arthritis and represents the prodromal stage of gout attack. Failure to quickly eliminate uric acid from purines causes several disruptions in the metabolic environment and may even harm the kidneys and liver (Bussler et al. 2017). It is also characterized by dysregulated renal excretion or aberrant hepatic metabolism, with a rise in blood uric acid content to 6–7 mg/dL (Chen‐Xu et al. 2019).

**Fig. 3:**
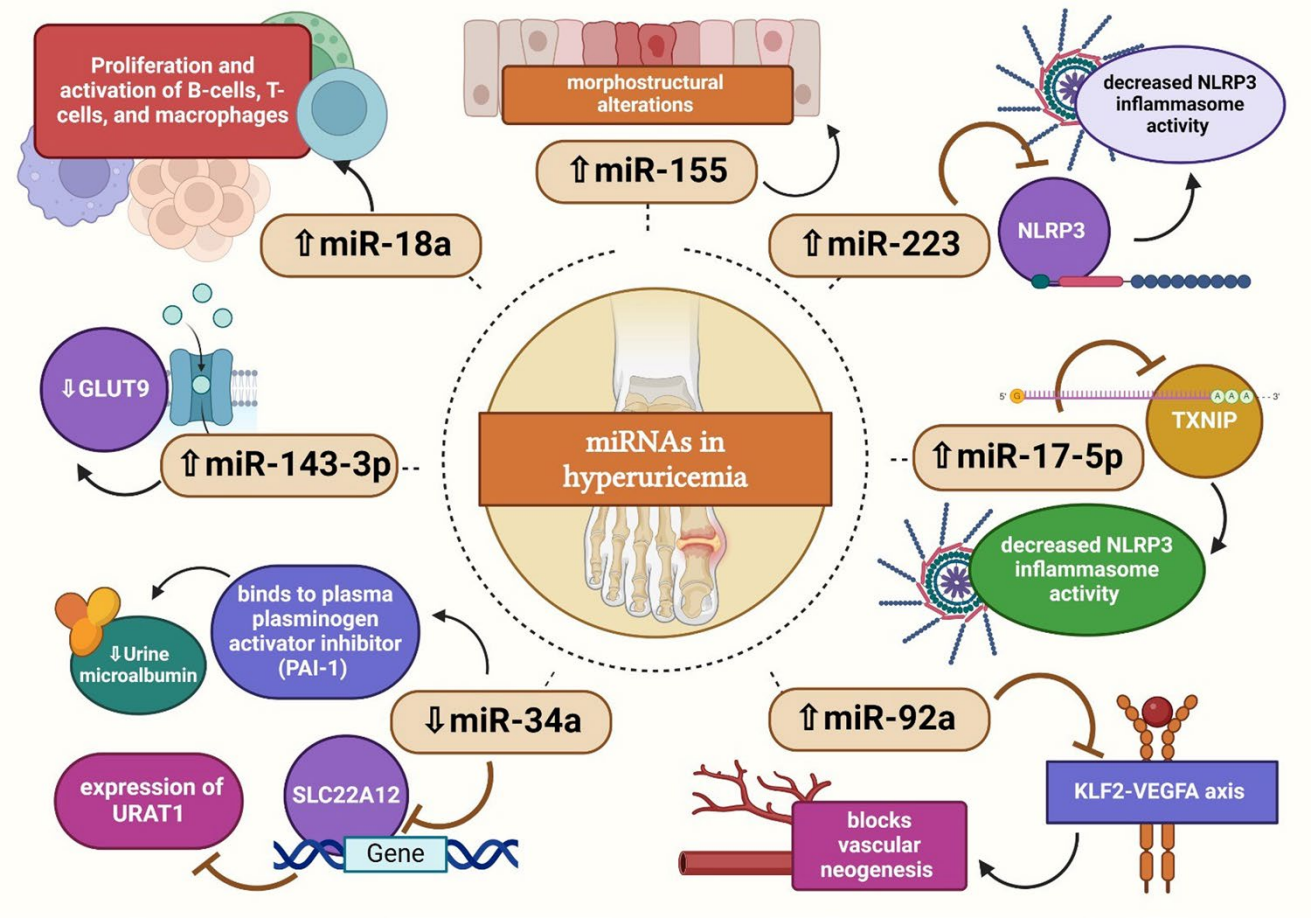
Role of miRNA in hyperuricemia. High levels of hsa-miR-155 are associated with morpho-structural alterations. hsa-miR-223 could inhibit NLRP3 expression, resulting in decreased NLRP3 inflammasome activity. hsa-miR-17 and hsa-miR-18a are involved in oncogenesis, proliferation, and activation of B-cells, T-cells, and macrophages. hsa-miR-17-5p hinders the activity of NLRP3 inflammasomes by attaching to and reducing the mRNA levels of thioredoxin-interacting protein. Decreased hsa-miR-92a inhibits vascular neogenesis by blocking the KLF2-VEGFA axis. hsa-miR-34a inhibits the expression of human URAT1. Low levels of hsa-miR-143-3p stimulate uric acid reabsorption. GLUT9: glucose transporter9; KLF2: Krüppel-like factor 2; NLRP3: NOD-like receptor protein 3; SLC22A12: solute carrier family 22 member 12; TXNIP: thioredoxin-interacting protein; URAT1: urate transporter 1; VEGFA: vascular endothelial growth factor A. This figure was created with BioRender (https://biorender.com/)

The hsa-miR-155 is implicated in several diseases (Abd-Elmawla et al. 2023a). In a clinical study, individuals with urate deposition showed higher levels of hsa-miR-155 than those without deposition findings, and the serum of HUA patients showed higher levels of hsa-miR-155 (Estevez‐Garcia et al. 2018). Elevated hsa-miR-155 levels were associated with morpho-structural alterations by ultrasonography that suggested the existence of urate deposits in both gout and asymptomatic hyperuricemia (Estevez‐Garcia et al. 2018) (Fig. 3).

It was previously shown that hsa-miR-223 is a myeloid-specific miRNA with the ability to inhibit NLRP3 expression, resulting in decreased NLRP3 inflammasome activity (Bauernfeind et al. 2012). Targeting and adversely regulating NLRP3 expression, hsa-miR-223 manages macrophage inflammasome activity (Bauernfeind et al. 2012). However, patients with gout, hyperuricemia, and gout flare-ups were shown to have upregulated hsa-miR-223. The presence of hsa-miR-223 upregulation in the plasma samples of those patients may suggest that inflammation persists even in the context of hyperuricemia (Bohatá et al. 2021). Increased inflammation generally leads to the upregulation of hsa-miR-223. This upregulation serves as a regulatory mechanism to modulate the immune response and control excessive inflammation by targeting several genes involved in inflammatory pathways (Neudecker et al. 2017). Although several papers reported the downregulation of hsa-miR-223 after treatment of cells with MSU, these papers were in vitro for a short period of time. The longest period in the mentioned studies was for 24 h, and this study showed a prompt increase of hsa-miR-223 levels in response to MSU, followed by downregulation until reaching a steady state, and the curve shows a tendency for gradual increase. Further investigation with longer periods to study fluctuations in hsa-miR-223 levels can explain the conflict between clinical and in vitro results as different expressions of hsa-miR-223 were found to be cell type and time-dependent (Tian et al. 2021; Wang et al. 2021; Yang et al. 2021).

hsa-miR-17 and hsa-miR-18a are part of the hsa-miR-17–92 cluster, which is involved in oncogenesis (He et al. 2005), proliferation, and activation of B-cells, T-cells, and macrophages (Kuo et al. 2019). Compared to the group of individuals with normal uric acid levels, there was a notable increase in the expression of hsa-miR-17 and hsa-miR-18a in both gout and hyperuricemia patient groups. There was also an observed elevation in the expression of hsa-miR-17 in those undergoing a gout flare-up. Likewise, hsa-miR-17-5p has been reported to hinder the activity of NLRP3 inflammasomes by attaching to and reducing the mRNA levels of thioredoxin-interacting protein (Lerner et al. 2012; Chen et al. 2018).

Likewise, the downregulation of hsa-miR-92a resulted from hyperuricemic stimulation, which blocked vascular neogenesis by blocking the KLF2-VEGFA axis (Yu et al. 2015). Conversely, in hyperuricemia-affected mice, mmu-miR-34a may suppress the expression of human URAT1 (Sun et al. 2011, 2015).

miRNAs can control the expression of genes related to urate transport. For instance, in animal models of hyperuricemia, mmu-miR-34a inhibits the production of URAT1 by targeting the mRNA of the SLC22A12 gene (Sun et al. 2015). A different study discovered that hsa-miR-34a binds to a plasma plasminogen activator inhibitor (PAI-1) to reduce urine microalbumin and enhance renal function (Liu et al. 2020).

Another uric acid transporter that controls uric acid excretion and reabsorption is called GLUT9 (Auberson et al. 2018). Hyperuricemia is caused by GLUT9 malfunction, as demonstrated by in vivo investigations. According to Zhou et al., the kidney tissue of hyperuricemic mice had considerably lower levels of mmu-miR-143-3p than the normal control (Zhou et al. 2019). By suppressing the expression of GLUT9, overexpression of mmu-miR-143-3p in vivo showed its ability to lower uric acid reabsorption. The kidney and intestine express ABCG2, also referred to as the ATP-binding cassette transporter, which is involved in the transport of medications and uric acid (Xu et al. 2020).

The above study is summarized in Fig. 3 and Table 1. In conclusion, miRNAs have a significant role in the development of hyperuricemia, and focusing on miRNA processing may offer fresh perspectives on how to manage the condition in the future.

**Table 1:** Role of miRNAs in gouty arthritis

miRNA	Biomarker role	Expression levels	Ref
hsa-miR-155	Morphostructural changes suggestive of urate deposits	↑↑	(Estevez-Garcia et al. 2018)
hsa-miR-223	Controls inflammasome activation in macrophages	↑↑	(Bauernfeind et al. 2012)
hsa-miR-17	Proliferation and activation of B-cells, T-cells, and macrophages	↑↑	(Lerner et al. 2012; Chen et al. 2018)
hsa-miR-18a	Proliferation and activation of B-cells, T-cells, and macrophages	↑↑	(Lerner et al. 2012; Chen et al. 2018)
hsa-miR-92a	Inhibiting vascular neogenesis	↓↓	(Yu et al. 2015)
hsa-miR-34a	Improves renal function	↓↓	(Sun et al. 2015)
hsa-miR-143-3p	Decreases uric acid reabsorption	↑↑	(Zhou et al. 2019)

It is essential to investigate the specific roles of miRNAs in modulating critical signaling pathways, such as the NLRP3 inflammasome and TLR4, which are integral to the inflammatory processes of gouty arthritis, building on the understanding of their impact on broader immunological and inflammatory responses.

## miRNAs modulate NLRP3 inflammasome and TLR4 signaling pathway

The NLRP3 inflammasome plays a crucial function in mediating inflammation in GA and it is composed of NLRP3, apoptosis-associated speck-like protein (ASC), and caspase-1. Pro-IL-1β can respond to damage-associated molecular patterns (DAMPs) that can bind to pattern recognition receptors (PRRs), leading to the recruitment of ASC that sequentially recruits and activates caspase-1 (Abd-Elmawla et al. 2023b). Finally, caspase-1 converts pro-IL-1β to mature IL-1β. On the other hand, the deactivation of NLRP3 and abolishment of IL-1 β exert a favorable effect on the mitigation of GA attacks. A panel of miRNAs was recognized as a potential modulator of the NLRP3 inflammasome, thus influencing GA pathogenesis (Sutterwala et al. 2014; He et al. 2016; Kelley et al. 2019).

Another key player implicated in the ongoing inflammation of GA is TLR. They are identified as a group of the PRR family that influences innate immune reactions. TLR4 identifies specific DAMPs and controls the inflammatory response. In GA, TLR-myeloid differentiation protein 2 (MD2) forms TLR4-MD2 complex, which recruits MyD88 and stimulates nuclear factor-κB (NF-κB), leading to an excessive rise of inflammatory cytokines such as IL-1β, IL-18, and TNF-α. However, the detailed mechanisms that modulate the NLRP3/TLR4 cascade are not fully elucidated (Kawai and Akira 2007; Sabroe et al. 2008).

Indeed, diverse miRNAs modulate the NLRP3 inflammasome and TLR4 signaling pathways, as shown in Table 2. Suppression of hsa-miR-192 and hsa-miR-561 stimulates TLR4 and NLRP3, which boost the inflammatory cytokines in GA (Lian et al. 2021). hsa-miR-192 possesses an emerging role in several disorders, such as liver and heart diseases (Nielsen et al. 2018; Sun et al. 2020). Another study discloses that the mmu-miR-23a-5p was high in animal models of GA. Elevated mmu-miR-23a-5p activates inflammation and the MyD88/NF-κB axis via activation of TLR2. Conversely, the abolishment of TLR2 mitigates the deleterious actions induced by miRNA-23a-5p (Li et al. 2022b). Interestingly, the role of mmu-miR-23a-5p was reported in tuberculosis infection through influencing TLR2/MyD88/NF-κB (Gu et al. 2017), whereas it could exert other oncogenic functions (Quan et al. 2017).

**Table 2:** miRNAs modulate the NLRP3 inflammasome and TLR4 signaling pathway in gouty arthritis

Ensembl	miRNAs	Location	Regulation	Target genes	Effect	Ref
ENSG00000283926	miR-192	Chr 11	↓↓	TLR4 NLRP3	Stimulates cytokine release and promotes inflammation	(Lian et al. 2021)
ENSG00000207951	miR-561	Chr 2				
ENSG00000207980	miRNA-23a-5p	Chr 19	↑↑	NF-κB MyD88 TLR2	Boosts inflammation	(Li et al. 2022b)
ENSG00000284567	miR-223	Chr X	↓↓	IL-1β TNF-α IL-37 TGF-β1	Inhibits IL-1β and TNF-α	(Zhang et al. 2021)
ENSG00000284043	miR-20b	Chr X	↓↓	NLRP3 IL-1β TNF-α	Activates NLRP3 and enhances inflammation	(Liu et al. 2021)
ENSG00000283904	miR-155	Chr 21	↑↑	SHIP-1 Akt NF-κB	Downregulates SHIP-1 and stimulates Akt/NF-κB Boosting inflammation	(Jin et al. 2014)
ENSG00000284567	miR-223-3p	Chr X	↓↓	NLRP3	Recruits NLRP3 inflammasome and promotes inflammation	(Wang et al. 2021)
ENSG00000283824	miR-22-3p	Chr 17				
ENSG00000265932	miR-3146	Chr 7	↑↑	SIRT1	Promote oxidative stress	(Shan et al. 2021)
ENSG00000215966	miR-876-5p	Chr 9	↓↓	NLRP3 TLR4 MyD88 NF-κB	Promotes inflammation	(Meng et al. 2021)
ENSG00000207870	miR-221-5p	Chr X	↓↓	TNF-α IL-8 IL-1β	Stimulates release of inflammatory markers	(Li et al. 2021)
ENSG00000283733	miR-146a	Chr 5	↓↓	TLR4 MyD88 NF-κB	Promotes inflammation	(Chen et al. 2019)
			↓↓	TRAK6 IRAK-1 NALP3	Promotes inflammation	(Zhang et al. 2018)
ENSG00000284463	miR-302b	Chr 4	↑↑	IL-1β NF-κB Caspase-1	Promotes inflammation	(Ma et al. 2018)
ENSG00000202609	miR-488	Chr 1	↓↓	IL-1β	Activation of IL-1β and promotion of inflammation	(Zhou et al. 2017)
ENSG00000216192	miR-920	Chr 12				

*Akt* protein kinase B, *IL-1β* interleukin-1 beta, *IL-8* interleukin 8, *IRAK1* IL-1 receptor-associated kinase, *MyD88* myeloid differentiation primary-response protein 88, *NALP3* leucine-rich repeat/pyrin domain-containing-3, *NLRP3* NOD-, LRR-, and pyrin domain-containing protein 3, *NF-κB* nuclear factor-κB, *TLR* toll-like receptor, *SHIP-1* Src homology 2 (SH2) domain-containing inositol-5-phosphatase 1, *SIRT1* silent information regulator sirtuin 1, *TGF-β1* transforming growth factor-β1, *TNF-α* tumor necrosis factor-alpha, *TRAF6* TNF receptor-associated factor 6

Previous research reported a low level of hsa-miR-223 in GA. Furthermore, hsa-miR-223 expression is restored after remission. The induction of hsa-miR-223 was markedly accompanied by the suppression of the NLRP3 inflammasome and cytokines such as IL-1β (Zhang et al. 2021). Relevantly, hsa-miR-223 possesses potential roles in the recruitment of immunological cells and proinflammatory mediators (Haneklaus et al. 2013). On the other side, hsa-miR-20b, which is located on chromosome Xq26.2, takes part in the pathological events of GA. A reciprocal relationship was detected between hsa-miR-20b and NLRP3 in synovial fluid mononuclear cells (SFMCs), where hsa-miR-20b was downregulated along with highly expressed NLRP3 in GA and MSU-stimulated THP-1 cells. Additionally, upregulation of hsa-miR-20b hinders inflammatory cytokines and deactivates NLRP3, which in turn mitigates GA flares (Liu et al. 2021). However, previous evidence declared that hsa-miR-20b modulated other inflammatory and immunological cascades as phosphatidylinositol-3-kinase/protein kinase B/mammalian target of rapamycin (PI3K/AKT/mTOR) pathway (İlhan et al. 2023).

Src homology 2 (SH2) domain-containing inositol-5-phosphatase 1 (SHIP1) deactivates phosphatidylinositol kinase (PIK) and cytokines. Moreover, SHIP1 suppresses ERK1/2, JNK, and NF-κB pathways (Boer et al. 2001; Kalesnikoff et al. 2002). It is noteworthy that SHIP-1 is implicated in several disorders, where it negatively regulates inflammatory and immune-mediated pathways (Maxwell et al. 2014). Wang et al. declared that the hsa-miR-223-3p and hsa-miR-22-3p were suppressed in gouty attacks whereas their induction attenuates the ongoing inflammation by hindering NLRP3 inflammasome (Wang et al. 2021). Regarding hsa-miR-155, it is implicated in several diseases, especially in GA patients. In addition, in vitro studies revealed its highly expressed regulation in MSU crystals, along with suppression of SHIP-1 and stimulation of the inflammatory mediators (Jin et al. 2014).

SIRT1 is considered one of the anti-uricemic agents, while deletion of SIRT1 aggravates GA attacks. SIRT1 plays these beneficial roles by suppressing diverse inflammatory pathways (Liu et al. 2019). The hsa-miR-3146 exerts a pronounced role in patients with elevated uric acid. Shan et al. (2021) elucidated that hsa-miR-3146 is highly expressed during gouty attacks along with triggered oxidative insults. hsa-miR-3146 exerts its inflammatory role by targeting SIRT1 (Shan et al. 2021). Interestingly, Chen et al. reported that hsa-miR-3146 modulates PTEN in liver cancer as well as glioma (Du et al. 2019; Qin et al. 2020). The hsa-miR-876-5p/NLRP3 cascade also participates in GA through modulating TLR4/MyD88/NF-κB signaling (Meng et al. 2021). However, hsa-miR-876-5p plays an important role in malignancies (Dong et al. 2019; Meng et al. 2021).

The hsa-miR-221-5p showed a low level in GA patients. Furthermore, IL-1β was reported as a direct target of hsa-miR-221-5p, and an inverse correlation was detected between them. Li et al. added that induction of hsa-miR-221-5p abolishes the rise of inflammatory markers such as TNF-α (Li et al. 2021). The mmu-miR-146a was downregulated in the GA model. Administration of miR-146a mimics relieved joint pain and inflammatory swelling, and the levels of TLR4 and MyD88 were decreased, followed by a marked decrease in the proinflammatory mediators (Chen et al. 2019). In the same direction, Zhang et al. (2018) added that the miR-146a abolishment triggers GA through the TRAK6/IRAK-1/NALP3 cascade (Zhang et al. 2018). This finding is in agreement with preceding studies, which declared that miR-146a exerts negative feedback action in inflammatory diseases through modulating NF-κB and attenuating the release of pro-inflammatory cytokines by targeting TRAF6 and IRAK1 (West and McDermott 2017).

Another report showed that in GA, miR-302b was elevated, which further modulates NF-κB, caspase-1, and IL-1β signaling (Ma et al. 2018). Moreover, miR-302b was identified as a marker for other pathological conditions (Zhang et al. 2014; Li et al. 2018). Zhou et al. showed that miR-488 and miR-920 were downregulated in the white blood cells of GA patients simultaneously with elevated IL-8 and TNF-α levels. Both miR-488 and miR-920 bind directly to the 3′-UTR of IL-1β (Zhou et al. 2017). Previous reports elucidated that the blockage of IL-1β release suppresses pain and ongoing inflammatory events. The activated IL-1β is elevated in hyperuricemic patients, which in turn recruits and activates other inflammatory and immunologic cells and mediators in the joints with the aid of TNF-α, which sequentially releases more cytokines. Song et al. added that miR-488 possesses a crucial function in chondrocyte differentiation and cartilage development (Song et al. 2013).

Knowing how miRNAs affect the TLR4 and critical inflammasome signaling pathways offers insight into the broader effects of miRNAs in gouty arthritis, especially bone erosion since osteoclast activity and bone resorption are greatly influenced by the inflammatory responses triggered by these pathways.

## miRNAs’ role in bone erosion

Gout is an inflammatory joint disease that is brought on by the buildup of crystals of monosodium urate (MSU) within the joints. Flares of gout can be intensely painful and severely impair a person’s quality of life (Chen-Xu et al. 2019).

In advanced cases of gout, joint damage and deformity can occur due to localized tophi-caused bone erosion and cartilage degradation. In gouty bone erosion, there is an imbalance between the production and resorption of bone (Luo et al. 2022). MSU crystal deposition triggers an inflammatory response and thus promotes the recruitment of neutrophils to the surface of osteoblasts (OBs) and their interaction with it through binding of neutrophil surface protein complex CD29/CD49d to osteoblast surface protein CS1, and then bone absorption by osteoclasts (Allaeys et al. 2011). An important route in the control of bone remodeling is the RANK/RANKL/OPG axis. RANKL is the essential component for osteoclast differentiation and activation, while OPG is the deceptive receptor of RANKL that downregulates bone resorption. In gout, MSU crystals inhibit the expression of osteoprotegerin (OPG), so the patients have higher circulating levels of RANKL. Thus, blocking RANKL activity is a potential strategy to lessen bone loss in GA patients (Chhana et al. 2016).

Numerous investigations have shown that miRNAs have a role in the onset of bone degradation.

Several miRNAs have been found to mediate osteoclast production and bone resorption through the PI3K/AKT and NF-κB signaling pathway; upregulated miRNAs include hsa-miR-let7e and hsa-miR-99b/132/148a/212, while downregulated miRNA include hsa-miR-29b (Hrdlicka et al. 2019). Inhibiting osteoclast development and bone resorption can be achieved by downregulating hsa-miR-31 (Mizoguchi et al. 2013). hsa-miR-20a may have a regulatory role in osteoclastogenesis. Thus, when it is upregulated, it reduces the expression of the RANKL gene through the TLR4/p38 pathway (Kong et al. 2021). hsa-miR-23a downregulates LRP5 expression, resulting in an elevation of calcium retention and a reduction in bone loss (Sujitha et al. 2020).

hsa-miR-17 has an anti-inflammatory and anti-erosive effect in vivo by specifically targeting JAK1 and STAT3, therefore inhibiting the IL-6 family’s autocrine actions (Najm et al. 2020). Mmu-miR-192-5p overexpression reduces EREG expression and improves bone erosion in MSU-induced GA mice (An and Yin 2021). hsa-miR-29b, which is one of the hsa-miR-29 family, regulates osteoclastogenesis. When hsa-miR-29b is upregulated, it can prevent human osteoclast differentiation by downregulating the expression of genes such as RANKL (Rossi et al. 2013). During osteoclastogenesis, hsa-miR-133a expression was elevated and promoted RAW264.7 and THP-1 cell differentiation into osteoclasts via RANKL (Wang et al. 2012). Also, the upregulation of hsa-miR-218 or hsa-miR-618 has been linked to a reduction in the development of RAW264.7 cells into osteoclasts, and this effect is mediated through the TLR-4/MyD88/NF-κB signaling pathway (Wang et al. 2017). The upregulation of hsa-miR-214 results in decreased PTEN levels, enhanced stimulation of the PI3K/Akt pathway, and ultimately improved osteoclast formation (Table 3) (Zhao et al. 2015).

**Table 3:** miRNA role in bone erosion

miRNAs	Alteration	Function	Reference
hsa-miR-let7e	↑↑	Mediate osteoclast formation and bone resorption through the PI3K/AKT, NF-κB signaling pathway	(Hrdlicka et al. 2019)
hsa-miR-99b			
hsa-miR-125a			
hsa-miR-132			
hsa-miR-148a			
hsa-miR-212			
hsa-miR-29b	↓↓		
hsa-miR-31	↓↓	Inhibits osteoclast formation and bone resorption	(Mizoguchi et al. 2013)
hsa-miR-20a	↓↓	Inhibit the differentiation of human osteoclasts	(Kong et al. 2021)
hsa-miR-23a	↓↓	Reduces bone loss and elevates calcium retention	(Sujitha et al. 2020)
hsa-miR-17	↑↑	Inhibits the autocrine effects of the IL-6 family	(Najm et al. 2020)
mmu-miR-192-5p	↑↑	Enhances bone erosion in MSU-induced GA mice	(An and Yin 2021)
hsa-miR-29b	↑↑	Inhibit the differentiation of human osteoclasts	(Rossi et al. 2013)
hsa-miR-133a	↑↑	Promote osteoclastogenesis	(Wang et al. 2012)
hsa-miR-218 hsa-miR-618	↑↑	Reduce osteoclastogenesis	(Wang et al. 2017)
hsa-miR-214	↑↑	Increase osteoclast formation	(Zhao et al. 2015)

*AKT* protein kinase, *IL-6* interleukin-6, *MSU* monosodium urate crystals, *NF-κB* nuclear factor kappa B, *PI3K* phosphatidyl inositol 3-kinase

Considering the pivotal function of miRNAs in facilitating bone erosion via their impact on osteoclast activity and inflammatory signaling, it is imperative to investigate their clinical relevance in gouty arthritis, where these molecular regulators may act as prospective biomarkers and therapeutic targets for disease progression and bone health management.

## Clinical importance of miRNAs in gouty arthritis

Firstly, miRNA is abundantly present in several bodily fluids, including the entire human blood (Backes et al. 2016), urine (Mall et al. 2013), and saliva (Ghizoni et al. 2020). Furthermore, miRNA exhibits stability in physiological fluids via a particular secretion mechanism, making it readily extractable from tissue samples without the need for invasive procedures. Despite fluctuations in environmental circumstances, miRNA may persist stably. Due to its high specificity, sensitivity, and consistent expression across many disorders, miRNA has the ability to provide early diagnosis with speed and accuracy (Witwer 2015).

Sixteen miRNAs expressed exclusively in GA patients were identified by Gupta et al. (Gupta and Elfiky 2019). The expression of miR-142-3p was significantly elevated in both in vivo and in vitro models of GA (Lu et al. 2022). In the plasma of GA patients, Bohatá et al. found elevated levels of numerous miRNAs, including hsa-miR-142, hsa-miR-17, hsa-miR-30c, hsa-miR-18a, and hsa-miR-223 (Bohatá et al. 2021).

**Fig. 4:**
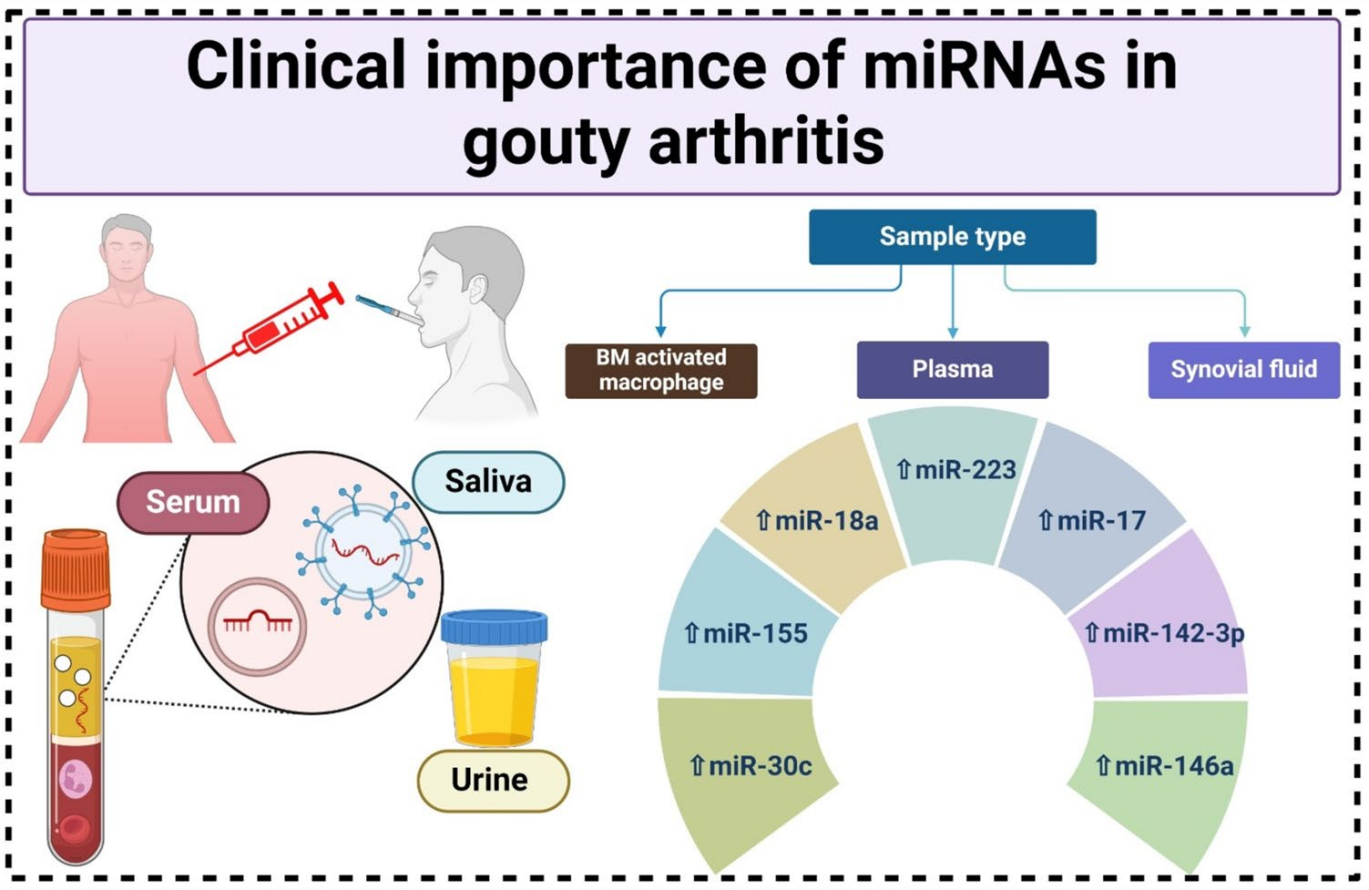
Clinical importance of miRNAs in gouty arthritis. The expression of miR-142-3p was significantly elevated in both in vivo and in vitro models of GA. Elevated levels of hsa-miR-142, hsa-miR-17, hsa-miR-30c, hsa-miR-18a, and hsa-miR-223 were detected in the plasma of patients diagnosed with GA. The miR-155 was detected in the mononuclear cells of synovial fluid. The level of mmu-miR-146a expression was elevated in mouse bone marrow-derived macrophages activated by MSU crystals. This figure was created with BioRender (https://biorender.com/)

Using a mouse model of gout and individuals with androgenetic alopecia, Jin et al. found elevated levels of miR-155 in the mononuclear cells of synovial fluid (Jin et al. 2014). After activating macrophages generated from mouse bone marrow with MSU crystals, Zhang et al. (2018) found that mmu-miR-146a expression was enhanced (Zhang et al. 2018). Hence, the atypical expression of the aforementioned miRNAs may assist in the prompt identification and management of GA and is anticipated to serve as a promising biomarker for GA diagnosis (Fig. 4).

Acknowledging the potential of miRNAs as therapeutic targets and biomarkers in gouty arthritis opens the door to a more thorough investigation of the underlying mechanisms that could be used to use these small RNA molecules to create effective treatment plans for this crippling illness.

## The mechanisms of miRNAs in the treatment of gouty arthritis

In the context of gouty arthritis, numerous studies have investigated the role of specific miRNAs in the development and progression of gouty arthritis, as well as their potential use as therapeutic agents. The following are some important findings.

hsa-miR-155 is a miRNA that has undergone thorough investigation into many inflammatory disorders (Li et al. 2022b). In gouty arthritis, hsa-miR-155 is upregulated in the synovial fluid and tissues of the affected joints. This upregulation suggests that hsa-miR-155 may contribute to the inflammatory response and disease progression in gout (Yang et al. 2018). hsa-miR-155 plays a crucial role in gouty arthritis by acting as a proinflammatory regulator via SH_2_ domain-containing inositol phosphatase 1 (SHIP-1) down-regulation and provoking the production of proinflammatory cytokines, such as TNF-α and IL-1β. This indicates a potential therapeutic target for treating acute gouty arthritis via the hsa-miR-155/SHIP-1 pathway (Jin et al. 2014). Moreover, research has shown that hsa-miR-155 can promote inflammation by targeting anti-inflammatory molecules such as the suppressor of cytokine signaling 1 (SOCS1), a negative regulator of pro-inflammatory cytokines (Jin et al. 2014). Therefore, inhibiting hsa-miR-155 enhances SOCS1 upregulation and may be involved in the treatment of gouty arthritis (He et al. 2017; Li et al. 2021).

hsa-miR-34a has been reported to regulate genes involved in uric acid metabolism, which is central to the development of gouty arthritis. hsa-miR-34a can target urate transporter 1 (URAT1), a key protein involved in renal uric acid reabsorption (Sun et al. 2015). Hence, modulating hsa-miR-34a levels may have an impact on urate levels and could potentially be explored as a therapeutic approach.

hsa-miR-449a is another miRNA that contributes to the therapy of gouty arthritis by targeting NLRP3 and epiregulin (EREG) to hinder the process of macrophage M1 polarization in gout. For instance, tripterine, a promising medicinal compound, has been studied for its role in improving the symptoms of gouty arthritis generated by MSU crystals by regulating the hsa-miR-449a/NLRP3 axis, which influences inflammatory processes. In addition, hsa-miR-449 has been observed to influence the expression of histone deacetylase 1 (HDAC1), which mediates the generation of cytokine production in MSU crystals (Liu et al. 2021).

Along with hsa-miR-449a, it specifically acts on NLRP3 and EREG to suppress the process of macrophage M1 polarization in gout, hence playing a role in controlling the development of the disease (Liu et al. 2021). In addition, hsa-miR-192-5p mitigated fibrosis and inflammatory responses by specifically targeting the nuclear factor of activated T cells (NFAT5), which is associated with the activation of macrophages and TLR-promoted arthritis (Jin et al. 2014), pointing toward the role of hsa-miR-192-5p in modulating disease processes.

hsa-miR-23a-5p has been stated to be essential in GA development and its therapy. The previous investigation indicates that hsa-miR-23a-5p enhances inflammation in gout by stimulating the myeloid differential protein-88 (MyD88)/nuclear factor-kappa B (NF-κB) pathway and inducing toll-like receptor-2 (TLR2), confirming the involvement of TLR2 inhibition in mitigating the inflammatory consequences of miRNA-23a-5p while GA treatment (Elseweidy et al. 2022).

hsa-miR-142-3p may exhibit dual functionality as an anti-inflammatory and pro-inflammatory gene in the context of inflammation. The role of hsa-miR-142-3p in promoting the inflammatory response in gouty arthritis is facilitated by binding with zinc finger E-box binding homeobox 2 (ZEB2) to activate the NF-κB signaling pathway (Li et al. 2022a). Overall, understanding the function of hsa-miR-142-3p in regulating proinflammatory cytokines is essential for the development of therapeutic therapies for GA.

hsa-miR-146a plays a significant role in reducing inflammation in acute gouty arthritis by acting on the TLR4/MyD88/NF-KB signaling pathway (Chen et al. 2019). In summary, even though hsa-miR-146a is linked to the etiology of GA, further research may be needed to establish its direct therapeutic applications in treating the condition. hsa-miR-3146 has a substantial impact on GA development by triggering the formation of neutrophil extracellular traps, which worsens gout flare-ups (Bergheim et al. 2008). The administration of antagomir-3146 has demonstrated contrasting effects, suggesting its potential as a viable target for therapeutic intervention (Shan et al. 2021).

hsa-miR-92a allows elevated uric acid levels to hinder the formation of new blood vessels by influencing the interaction between Krüppel-like factor 2 (KLF2) and vascular endothelial growth factor-A (VEGFA). hsa-miR-92a specifically interacts with KLF2, which then attaches to the VEGFA promoter, hence controlling the process of angiogenesis. This interaction emphasizes the intricate molecular processes associated with the suppression of angiogenesis by uric acid (Yu et al. 2015). Gaining insight into these pathways could aid in the development of precise treatments for illnesses associated with abnormal blood vessel formation.

**Fig. 5:**
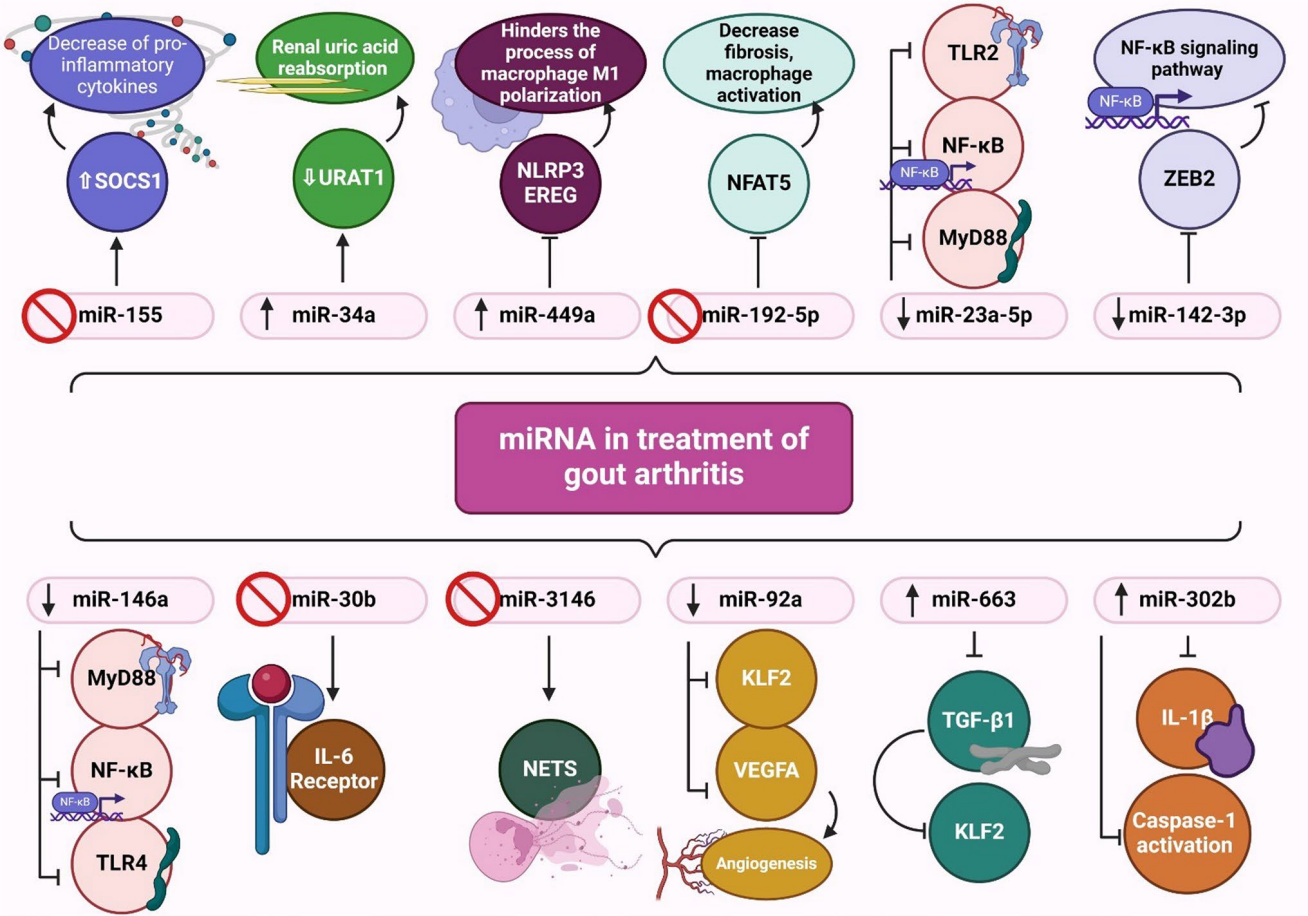
Role of miRNA in treatment of gout arthritis. hsa-miR-155 acts as a proinflammatory regulator via SH2 domain-containing inositol phosphatase 1 (SHIP-1) down-regulation and provokes the production of proinflammatory cytokines, such as TNF-α and IL-1β. hsa-miR-155 promotes inflammation by targeting anti-inflammatory molecules such as SOCS1, a negative regulator of pro-inflammatory cytokines. hsa-miR-34a modulation may have an impact on urate levels and could potentially be explored as a therapeutic approach. hsa-miR-449a influences the expression of HDAC1, which mediates the generation of cytokine production in MSU crystals. hsa-miR-192-5p targets NFAT5which is associated with the activation of macrophages and TLR-promoted arthritis. hsa-miR-23a-5p enhances inflammation in gout by stimulating MyD88/NF-κB. hsa-miR-142-3p promotes the inflammatory response by binding with ZEB2 to activate the NF-κB signaling. hsa-miR-146a acts on the TLR4/MyD88/NF-KB signaling pathway. hsa-miR-3146 triggers the formation of neutrophil extracellular traps. hsa-miR-92a influences KLF2/ VEGFA. hsa-miR-663 interacts with TGF-β1 to target and control the expression of PTEN. hsa-miR-302b exerts a negative regulatory effect on the production of IL-1β. hsa-miR-488 and hsa-miR-920 have the capability to directly bind to the 3′ untranslated region (UTR) of IL-1β and particularly alter its function. hsa-miR-223-3p and hsa-miR-22-3p decrease the synthesis of IL-1β by specifically targeting NLRP3. hsa-miR-221-5p has been detected to greatly suppress the expression of TNF-α, IL-8, and IL-1β. EREG: epiregulin; IL-1β: interleukin-1 beta; IL-6: interleukin-6; KLF2: Krüppel-like factor 2; MyD88: myeloid differentiation primary-response protein 88; NETs: neutrophil extracellular traps; NFAT5: nuclear factor of activated T-cells 5; NF-κB: nuclear factor kappa B; SOCS1: suppressor of cytokine signaling 1; TGF-β1: transforming growth factor-β1; TLR2: toll-like receptor 2; TLR4: toll-like receptor 4; URAT1: urate transporter 1; VEGFA: vascular endothelial growth factor A; ZEB2: zinc finger E-box binding homeobox. This figure was created with BioRender (https://biorender.com/)

By enhancing its activity, hsa-miR-663 helps uric acid impede the movement of endothelial cells. This molecule specifically interacts with transforming growth factor-β1 (TGF-β1) to target and control the expression of phosphatase and tensin homolog (PTEN). This mechanism involves hsa-miR-663 suppressing TGF-β1, which subsequently affects PTEN, ultimately suppressing the migration of endothelial cells (Yu et al. 2015). hsa-miR-302b exerts a negative regulatory effect on the production of IL-1β, a crucial proinflammatory cytokine that is closely linked to gout inflammation. By specifically focusing on interleukin-1 receptor-associated kinase 4 (IRAK4) and ephrin type-A receptor 2 (EphA2), hsa-miR-302b may behave as a potentially effective therapeutic strategy for suppressing inflammation mediated by NF-κB and caspase-1 activation in GA (Auberson et al. 2018). Meanwhile, other miRNAs have a role in regulating IL-1β, which in turn has a notable influence on GA. Specifically, hsa-miR-488 and hsa-miR-920 can directly bind to the 3′ untranslated region (UTR) of IL-1β and particularly alter its function (Zhou et al. 2017). Similarly, hsa-miR-223-3p and hsa-miR-22-3p decrease the synthesis of IL-1β by specifically targeting NLRP3, therefore mitigating the inflammatory reaction associated with gout (Kong et al. 2021). Moreover, the excessive expression of hsa-miR-221-5p has been detected to greatly suppress the expression of TNF-α, IL-8, and IL-1β (Fig. 5) (An and Yin 2021).

## The interplay of GA-associated miRNAs in personalized medicine and chronobiology

Personalized medicine integrates genetic data with phenotypic and environmental factors to produce patient-specific medical services and eliminates the boundaries of the “one-size-fits-all” treatment strategy. This offers the chance to transfer medications from the lab to the clinic, identify and prognosticate illness, enhance individualized tailored therapy according to the distinct signatures of a patient’s illness, and also discover new treatment plans (Chan and Ginsburg 2011).

Whole genome research revealed above 40 genetic loci that control blood uric acid levels. These genes modulate drug effects and side effects of gout therapy. The present advancements in epigenetic factors such as miRNAs offer the possibility of individualized management of GA according to their miRNA signatures (Yan and Zhang 2020). Currently, the miRNAs have received a great deal of interest due to their widespread participation in cellular processes either physiological or pathological. The individual’s variability in the expression of these miRNAs aids in the arising of personalized medicine (Dreussi et al. 2012). In other words, the total heterogeneity exhibited by these short non-coding RNAs influences the pharmacokinetics and pharmacodynamics of medications in a patient-specific manner, hence driving an increasing demand for individualized tailored therapy. Realizing the miRNA profile of each patient in GA enables its treatment using tailored medicine and better outcomes (Avci and Baran 2014; Latini et al. 2019).

Over and beyond, preceding studies revealed that GA attacks happen more often at night or in the early morning, however, the underlying reasons have not been recognized till now. These findings highlight the potential benefits of studying gout’s circadian fluctuation to achieve the optimal timing of anti-gout preventive treatments (Ursini et al. 2021). Simultaneously, ample evidence disclosed miRNAs as potential modulators of circadian rhythmicity, providing a novel approach to the management of GA according to the biological clocks. miRNA pertubations are implicated in Circadian rhythm disruption which sequentially results in various inflammatory diseases such as GA. In the era of personalized medicine, miRNA-based therapeutic strategies should be time-dependent to achieve the most significant efficacy (Pegoraro and Tauber 2008; Škrlec 2023).

In the next years, it is expected that the expanding information about miRNAs will find broad clinical applications, especially in the development of personalized medicine. Consequently, it is mandatory to dig deeper into the biological behavior, and clinical consequences of these miRNAs as previously discussed in the current review in an attempt to identify the distinct miRNA profiles in GA that may yield better therapeutic strategies and improve patients’ quality of life (Yan and Zhang 2020).

## Conclusion

Recently, miRNAs have received significant interest in the field of biological research. Recent research has demonstrated a strong correlation between miRNAs and the advancement of gouty arthritis. Additionally, miRNAs play an important role in the control of genes after transcription. Although numerous studies have documented miRNA’s role in regulating the inflammatory process of gout, there is a notable deficiency in understanding its impact on gouty arthritis, particularly bone degradation. The precise mechanisms behind the spontaneous resolution of gout, a recurring chronic condition, and the development of gouty stones are still not fully understood. Neutrophil-associated NETosis refers to a process in the inflammatory response when neutrophils release extracellular traps. It is unclear if this mechanism exacerbates or relieves inflammation and whether it contributes to repeated gout attacks. Despite the availability of treatments for gout, the medications used still have noticeable negative effects. Targeting miRNAs could offer a fresh perspective and a potential solution for treating gout. In the future, miRNA is anticipated to serve as a diagnostic marker for gouty arthritis or as a target for pharmaceutical intervention. Nevertheless, additional research is still required. Hence, it is imperative to investigate and comprehend the mechanisms of key miRNAs to accurately diagnose and effectively treat gouty arthritis.

## Data Availability

All source data for this work (or generated in this study) are available upon reasonable request.
